# International Congress of the Medical Sciences

**Published:** 1878-01

**Authors:** 


### International Congress of the Medical Sciences. Held at Geneva, Switzerland, Sept. 9-15, 1877.

### FIFTH BIENNIAL SESSION.

The opening session was held in the Aula of the University, at three o'clock, p. m., on Sunday, September 9th.

The President of the Committee of Organization, M. Carl Vogt, gave the opening address. Federal Counsellor, Numa Dorz, then pronounced an eloquent discourse, in which he eulogized the medical sciences and gave a warm welcome to the delegates. MM. Carterel and Rivoire, on the part of the municipal government, followed in welcoming the representatives of other countries to the city of Geneva.

The addresses of welcome being over, Carl Vogt then made a formal opening of the business of the session by a discourse upon the question: Is Medicine an Exact Science in the same sense as Mathematics, Physics, etc.? To this the learned speaker was compelled to return a negative answer. He endeavored to show that this is due, in a great measure, to the impossibility of making experiments upon human life and health, to the uncertainty of conclusions drawn from experiments upon the lower animals; then he proceeded to criticise the lack of method and true scientific spirit among medical men, and, finally, the defectiveness in their preparatory education. Much of the blame he was disposed to put upon the great amount of time devoted to classical studies.

The General Secretary, Prof. Prevost, then read his report upon the organization of the Congress. The provisional organization was made permanent, and, on motion of Prof. Hardy, of Paris, Honorary Presidents were chosen from the different countries represented, among them, Drs. J. Marion Sims and E. Seguin, for the United States. The session then closed.

In the evening an entertainment was given in one of the public buildings, enlivened by music and graced by the presence of many fair ladies, who did the honors, as was to be expected, in a manner at once enchanting and unexceptionable.

Second Day, Monday, Sept. 10.—The Sections met in the morning, the general meeting was held at three p. m., M. H. Lombard presiding. Dr. Broadbent, of London, read his report upon Cerebral Localizations. We give a short abstract of his conclusions:

1. On the Motor Zone of the Cerebral Cortex:

*a.* Physiological experiments have established the fact that there exists a certain zone in the cortical portion of the cerebral hemispheres which is in intimate relation with the nuclei of the motor nerves in the medulla oblongata and the spinal cord. In the monkey and in man this zone is situated about the fissure of Rolando, principally in the two ascending or marginal convolutions, which bound this fissure. In this region there exist certain tracts related, more or less definitely, to the movements of the face, arm, leg, etc.

*b.* The pathology of this region is in remarkable accord with the results of experimentation. Partial and irritative lesions produce partial epilepsy, with or without transient or permanent hemiplegia. More extensive lesions, with more or less extensive destruction of the gray cortical substance, cause monoplegia or hemiplegia.

*c.* While admitting this localization of motor centers he also assumes that their connection with the anterior horns of the gray substance of the spinal cord is not direct, but takes place through intermediate ganglia. He concludes that the motor centers of the cerebral cortex are the points of departure for ideo-motor actions, of voluntary impulses. The cells of the motor zone are the apparatus by which the dictates of the intelligence are formulated for transmission or outward expression.

*d.* According to the experiments of Hitzig and Ferrier, there exists in the cortical layer a region or zone for perception. This has not been demonstrated by pathology.

*e.* Certain portions of the brain have not been yet ascertained to be the seat of special functions. (We must refer our readers to

Ferrier on the Functions of the Brain, for a more extended discussion of these points, as also of the functions of the opto-striate bodies.)

*f.* There does not appear to be any cerebral vaso-motor center; the vaso-motor apparatus is in relation with the general motor system, and, like it, has centers in the cord, the central ganglia and in the convolutions. The same may be said of trophic nerves and trophic centers. Trophic influence resides in the entire nervous system.

During the discussion of Dr Broadbent's paper. Prof. Schiff raised some objections to the theories of the learned Englishman.

Dr. H. Cl. Lombard, of Geneva, read a report on Medical Geography, with special reference to Malaria in Europe and in North America, after which the general session adjourned.

THIRD DAY, TUESDAY, Sept. 11.—Prof. Schiff made a report on the Functions of the Spleen. His conclnsions were drawn from extirpation of that organ in animals; the most important of which are as follows:

1. The spleen, during digestion, especially during stomachic absorption, prepares the ferment which, entering with the blood into the tissue of the pancreas, transforms a special substance (probably albuminoid) into *pancreatopepsin* or trypsine, a substance capable of digesting albuminoid bodies.

2. After extirpation of the spleen, the pancreatic juice loses its digestive influence upon albuminoid bodies,while it preserves its other digestive properties. Duodenal digestion of albuminoids, normally distinguished by its energy and rapidity, then becomes as feeble as in any other part of the small intestine.

Prof. Hardy then read a work entitled: Some Considerations upon the Etiology,Nature and Treatment of Contagious Diseases of the Hairy Surface. The author dealt exclusively with parasitic affections; fatus, trichopta and pelade. In his opinion all these affections are of parasitic origin, only the last named being disputed All are eminently contagious, but every one does not offer the same susceptibility to contagion. Physical and moral depression have a great part in determining each person's liability to receive it.

FOURTH DAY, WEDNESDAY Sept. 12.—M. Bernhard, of Paris, read a report on the Causation of Typhoid Fever. He thought that the disease might be conveyed through the medium of the air and drinking water. He directed especial attention towards these means of infection. Contagion, he thought, occurred only exceptionally.

SESSION OF THURSDAY, Sept. 13th.—M. Lebert read a work on Chronic Ulcer of the Stomach. This was based on 252 cases observed by himself. His conclusions offer little novelty. In treating these cases he insists upon the efficacy of the milk diet.

M. Marey gave a remarkable lecture on the electrical discharges of the torpedo (cramp fish), which was received with much enthusiasm.

He demonstrated that there was a strong analogy betwen ordinary muscular contraction and the electrical discharges of this fish.

M. Gellé, of Brussels, discussed at great length the necessity for an Universal Pharmacopœia.

Dr. E Seguin, of New York, strongly advocated some uniform method of clinical observation in every country. It was decided to appoint a committee to report to the next Congress upon an Universal Pharmacopœia. Among others, Drs. J. Marion Sims and E. Seguin were appointed upon this committee at the session of Sept. 14th.

Dr. C. Vogt, at this session, made his report on the Entozoa of Man.

Seventh Day, Saturday, Sept. 15.—It was decided the will of the Congress to hold its next (sixth) session in Holland.

Carl Vogt then made the closing address and declared the Congress at an end. Dr. Pacchioili, of Turin, and Dr. Bouchard, of Paris, delivered addresses of thanks to the officers of the Congress and to the Swiss Federal and Cantonal authorities, for their cordial hospitality.

### SECTION OF MEDICINE.

First Session, Sept. 10.—Prof. Baccilli, of Rome,read a paper, in Latin, on a New Method of Treating Certain Aortic Aneurisms. This method is to be applied only to aneurisms by lateral dilatation, and puncturing the sac where it is most thin, with a capillary trocar and introducing through this puncture a watch spring. The watch spring becomes a formative center for blood-clots which eventually occlude the tumor. He reported several cases successfully treated by this method.

Dr. Proust reported a case of acute myelitis, in the name of Dr. Jeoffroy and himself. This case was interesting for its mode of onset, which had been a fall. Some of those who discussed the case thought the fall merely the first symptom of pre-existing disease.

Dr. Proust read the conclusions contained in a memoir on Phthisis, by Dr. Grancher. From clinical observation and pathological anatomy, the author recognized three forms of phthisis: common, ordinary phthisis, caseous pseudo-lobar pneumonia, and acute ulcerative phthisis. In his opinion caseous pneumonia and granulation do not exist without tubercle.

Second Day, Sept. 11.—Dr. Revilleod, of Geneva, read a long paper on Diphtheria. His views offer little that is new. We may state, however, that he thinks croup and diphtheria, to be identical; that there is no specific for the disease; that tracheotomy should be performed in the third state of croup, and that two-thirds of these cases ought to recover.

Prof. Hayem, of Paris, read one of the most important papers of this Congress, on the Alterations of theBlood in Anæmia. According to Hayem, all treatment is useless if iron is not taken by the patient.

THIRD DAY, Sept. 12.—M. Schnitzler, of Vienna, read an essay on the Employment of Compressed Air in diseases of theLungs and Heart, and M. Gimbert, of Cannes, on Treatment of Phthisis by Creosote. M. Carville thought Dr. Gimbert ought to change the title of his paper to Expectant Treatment of Phthisis. He thought the great physician of the South, the Sun, was entitled to the credit of the cures reported by Dr. Gimbert, and that every physician could obtain the same results by feeding his patient well.

FOURTH SESSION, Sept. 14.—Dr Cérenville, of Lausanne, read a paper on the Influence of Salicylic Acid over some of the Nervous Complications of Typhoid Fever,and the anti-pyretic action of that drug. Dr. Valcourt, of Cannes, reported two cases of death from Asphyxia by Compression of the Trachia by Diseased Bronchial Glands.

SUPLEMENTARY SESSION of Sept. 15.—M. Dejerine read, in the name of Dr. Vidal, physician to the St. Louis Hospital, Paris, the latter's conclusions on the Inoculability of Divers Skin Diseases. He has found ecthyma inoculable upon healthy persons, also auto-inoculable to the fourth and fifth generation of pustules.

Common herpes is, exceptionally, inoculable on the subject of the disease, herpes zoster is never inoculable, perputial herpes he has never seen inoculable.

Impetigo, and mentagra are auto-inoculoble. Pemphigus of the new-born (non-syphilitic) is auto-inoculable to the second and third eruption, upon the bearer. Eczema, pemphigus diutinus, and molluscum contagiosum (acne variolaformis) are never inoculable.

Dr. Debout d'Estrées followed with a communication on Hairy Gravel. His conclusions are as follows:

A species of gravel exists which is characterized by the emission of hairs with the urine. These hairs are sometimes accompanied by bony and cartilaginous tissues, which may be due to a fœtal cyst communicating with the urinary passages. At other times they are accompanied solely by uric acid and arise from an abnormal production of hair upon the mucous membrane of the urinary tract.

M. d'Espine read, in the name of Dr. Sangalli, a note on a case of perforation of the intestine by round worms.

A discussion followed on the use of the thermo-cautery in tracheotomy, after which the Section adjourned *sine die.—St Louis Record.*

